# Targeted Next-Generation Sequencing of Thymic Epithelial Tumours Revealed Pathogenic Variants in *KIT*, *ERBB2*, *KRAS*, and *TP53* in 30% of Thymic Carcinomas

**DOI:** 10.3390/cancers14143388

**Published:** 2022-07-12

**Authors:** Adam Szpechcinski, Malgorzata Szolkowska, Sebastian Winiarski, Urszula Lechowicz, Piotr Wisniewski, Magdalena Knetki-Wroblewska

**Affiliations:** 1 Department of Genetics and Clinical Immunology, The Institute of Tuberculosis and Lung Diseases, 01-138 Warsaw, Poland; u.lechowicz@igichp.edu.pl; 2 Department of Pathology, The Institute of Tuberculosis and Lung Diseases, 01-138 Warsaw, Poland; 3 Clinics of Thoracic Surgery, The Institute of Tuberculosis and Lung Diseases, 01-138 Warsaw, Poland; s.winiarski@igichp.edu.pl; 4 Department of Pathology and Laboratory Medicine, The Maria Sklodowska-Curie National Research Institute of Oncology, 02-781 Warsaw, Poland; piotr.wisniewski@pib-nio.pl; 5 Department of Lung Cancer and Chest Tumours, The Maria Sklodowska-Curie National Research Institute of Oncology, 02-781 Warsaw, Poland; magdalena.knetki-wroblewska@pib-nio.pl

**Keywords:** thymic epithelial tumours, thymoma, thymic carcinoma, next-generation sequencing, single nucleotide variants, *TP53*, *ERBB2*, *KIT*, somatic variants, germline variants

## Abstract

**Simple Summary:**

The biology of thymic epithelial tumours (TETs), including thymomas and thymic carcinomas, and particularly the extent of molecular dysregulation, is poorly understood. Through next-generation sequencing of 15 genes implicated in common solid tumours in 53 TETs, we found a larger number of single nucleotide variants (SNVs) in thymic carcinomas than thymomas. About 30% of thymic carcinomas had at least one somatic pathogenic gene variant in *TP53*, *ERBB2*, *KIT*, or *KRAS*, whereas variants of uncertain clinical significance in *KIT*, *ERBB2*, and *FOXL2* were found exclusively in thymomas. The presence of somatic pathogenic variants was non-significantly associated with shorter disease-free survival in thymic carcinoma patients. No somatic pathogenic or likely pathogenic SNV was found in thymomas. Importantly, we also evaluated germline SNVs, adding to the number of known genetic alterations in TETs and thereby enhancing our molecular understanding of these neoplasms.

**Abstract:**

A better understanding of the molecular pathogenesis of thymic epithelial tumours (TETs) could revolutionise their treatment. We evaluated thymomas and thymic carcinomas by next-generation sequencing (NGS) of somatic or germline single nucleotide variants (SNVs) in genes commonly mutated in solid tumours. In total, 19 thymomas and 34 thymic carcinomas were analysed for nonsynonymous SNVs in 15 genes by targeted NGS (reference genome: hg19/GRCh37). Ten SNVs in *TP53* (G154V, R158P, L194H, R267fs, R273C, R306 *, Q317 *), *ERBB2* (V773M), *KIT* (L576P), and *KRAS* (Q61L) considered somatic and pathogenic/likely pathogenic were detected in 10 of 34 (29.4%) thymic carcinomas. No somatic SNVs confirmed as pathogenic/likely pathogenic were found in thymomas. Rare SNVs of uncertain or unknown functional and clinical significance, to our knowledge not reported previously in TETs, were found in *ERBB2* (S703R), *KIT* (I690V), and *FOXL2* (P157S) in 3 of 19 (16%) thymomas. The most frequent germline SNVs were *TP53* P72R (94% TETs), *ERBB2* I655V (40% TETs), and *KIT* M541L (9% TETs). No significant difference in median disease-free survival (DFS) was found between thymic carcinoma patients with and without pathogenic SNVs (*p* = 0.190); however, a trend toward a longer DFS was observed in the latter (16.0 vs. 30.0 months, respectively). In summary, NGS analysis of TETs revealed several SNVs in genes related to the p53, AKT, MAPK, and K-Ras signalling pathways. Thymic carcinomas showed greater genetic dysregulation than thymomas. The germline and rare SNVs of uncertain clinical significance reported in this study add to the number of known genetic alterations in TETs, thus extending our molecular understanding of these neoplasms. Druggable KIT alterations in thymic carcinomas have potential as therapeutic targets.

## 1. Introduction

Thymic epithelial tumours (TETs) are rare malignant neoplasms of the prevascular mediastinum that account for approximately 0.2–1.5% of all malignancies and have an annual incidence of 1.3–3.2 cases per million [1,2]. TETs are histologically highly heterogeneous neoplasms. According to the 2021 World Health Organization (WHO) histological classification [3], this group includes thymomas and thymic carcinomas. Thymomas are characterised as low- or medium-grade malignancies, with a 5-year survival rate of >80% [4]. Thymic carcinomas are more aggressive, metastasise, and readily recur; the 5-year survival rate is about 36% [5]. Less advanced TETs (TNM stages I–IIIA) are treated surgically, and complete resection is of key prognostic importance. Locally advanced tumours (TNM stage IIIA/IIIB/IVA) require neoadjuvant treatment (chemotherapy or radiotherapy), and surgical reassessment. In cases of disseminated cancer, palliative platinum-based chemotherapy is the treatment of choice. Recurrent TETs are not uncommon and require management like primary tumours [6].

Personalised medicine based on genetic or epigenetic abnormalities in cancer cells, which is important in treatment of lung carcinoma, for example, does not yet play a key role in the treatment of TETs. Research into clinically significant molecular alterations is hampered by the rarity of the tumours, their clinical and biological heterogeneity, changes in histopathologic classifications, and a lack of well-established cell lines and animal models of these neoplasms. Recurrent gene mutations in thymomas and thymic carcinomas have been identified by next-generation sequencing (NGS), but their role in TET pathogenesis is unclear [7].

Advances in knowledge of the actionable molecular alterations and pathways involved in TET pathogenesis have enabled identification and evaluation of targeted therapies. Promising data have been reported regarding the activity of new targeted therapies for patients with TETs, particularly those harbouring specific gene alterations, including receptor tyrosine kinase inhibitors (*KIT*, *IGF1R*), anti-angiogenic drugs (*VEGR*, *FGFR*, *PDGFR*), and inhibitors of PARP (*BAP1*), cyclin-dependent kinases (*CDK*, *RB*), and PI3K/mTOR (*PI3K*) [7,8]. Better understanding of genomic alterations in TETs could revolutionise treatment of these tumours by facilitating the development of newer therapies, thus improving clinical outcomes. Further studies taking account of TET type and stage are needed to validate the predictive value of candidate biomarkers for biological, pathological, and clinical outcomes.

In this study, we evaluated thymomas and thymic carcinomas by targeted NGS of single-nucleotide variants (SNVs) in genes commonly mutated in solid tumours. The objective was to assess the frequency of *KIT* gene alterations, and other relevant somatic and germline genetic variants, in a Polish cohort of TET patients, and to correlate the genetic findings with the pathological and clinical features of these tumours.

## 2. Materials and Methods

### 2.1. Patient Cohort

This retrospective study included 53 patients (37 men and 16 women) aged 22–87 years (median age, 57 years) diagnosed and treated at our institutions between 2007 and 2021. All the patients were Caucasian. Five patients with combined B2B3 thymoma presented with symptoms of myasthenia gravis; no other paraneoplastic autoimmune diseases were found. The demographic and clinical characteristics of the patients are listed in Table 1. The study protocol was approved by the Institutional Review Board (no. KB-55/2021).

### 2.2. Tissue Samples

We included all thymic carcinomas diagnosed in the study period, and a representative group of thymomas of different histological subtypes and stages. Specimens of TETs were obtained by surgical resection or surgical biopsy performed during mediastinoscopy. Tumour histological subtype was determined by two experienced thoracic pathologists (M.S. and P.W.) according to the 2021 WHO classification and staged according to the eighth edition of the TNM system. Archival cases were reclassified in accordance with these classifications [3,9,10]. To simplify the analysis, cases of large cell neuroendocrine carcinoma, which is a separate subgroup of neoplasm in the WHO classification, were included in the thymic carcinoma group. The histopathological diagnosis in all thymic carcinomas (resections and biopsies), and in surgical biopsies of thymomas, was supported by an appropriate panel of immunohistochemical tests for each case. Resected thymomas generally did not require additional testing.

Formalin-fixed and paraffin-embedded (FFPE) archival tissue samples were immunohistochemically assessed for CD117 expression (clone EP10; Ventana Medical System Inc., Tucson, AZ, USA). The reaction was performed on one representative 4-µm-thick section from each tumour using a BenchMark Ultra Automated Immunostainer (Ventana Medical System Inc., Oro Valley, AZ, USA) according to the manufacturer’s recommendations. A membrane or cytoplasmic colour reaction in >1% of neoplastic cells was considered positive.

### 2.3. DNA Extraction

Five 8-μm-thick sections were cut from FFPE tissue blocks containing 50–95% cancer cells and collected in sterile Eppendorf tubes. DNA was extracted from the FFPE tumour tissue sections using the MagCore Genomic DNA FFPE One-Step Kit and MagCore HF16 Plus Automated Nucleic Acid Extractor (RBC Bioscience Corp., Taipei City, Taiwan), in accordance with the manufacturer’s instructions. The DNA concentration was assayed by UV spectrophotometry using the NanoVue Small Sample Volume Spectrophotometer (GE Healthcare UK Limited, Little Chalfont, UK) and by fluorimetry using the QuantiFluor ONE dsDNA System and Quantus Fluorometer (Promega, Madison, WI, USA), in accordance with the manufacturers’ protocols and laboratory guidelines [11,12].

### 2.4. NGS Analysis of SNVs

SNVs in 15 genes commonly mutated in solid tumours were detected by targeted NGS of the genes listed in Supplementary Table S1. Briefly, DNA libraries were constructed using the TruSight Tumor 15 assay (Illumina, San Diego, CA, USA). The concentration of each NGS library was measured by fluorimetry using the QuantiFluor ONE dsDNA System and Quantus Fluorometer (Promega). A quality check of the NGS libraries was performed by resolving an aliquot of each normalised library on 2% agarose gel with a 50 bp DNA ladder. Libraries meeting the qualitative and quantitative requirements were sequenced on the MiSeq instrument (Illumina) using the high-output MiSeq Reagent Kit v. 3 (Illumina) (2 × 150 bp read length) with dual indexing. This technique allows for sensitive and accurate detection of somatic variants with a 5% allele frequency, using 20 ng DNA with minimum coverage of 500×.

### 2.5. Bioinformatics and Computational Analysis of NGS Data

Processing of the NGS data, including demultiplexing, alignment and variant calling, was performed using MiSeq Reporter v. 2.6 software (Illumina). The resulting gVCF files (*.genome.vcf) were analysed using the BaseSpace Variant Interpreter application (Illumina) for variant annotation with the reference genome hg19/GRCh37.

Detected variants were analysed in terms of their coding region (missense, nonsense, or frameshift mutation), functional effects (gain or loss of function), and clinical significance (pathogenic, likely pathogenic, benign, likely benign, or uncertain) using the Sorting Intolerant from Tolerant (SIFT) algorithm [13], PolyPhen-2 prediction algorithm [14], Catalogue of Somatic Mutations in Cancer (COSMIC), v. 95 [15], ClinVar database [16], VarSome database [17], and International Cancer Genome Consortium (ICGC) Data Portal, release 28 [18]. The origin (somatic or germline) of the SNVs was evaluated according to previously reported data (COSMIC, ClinVar, and VarSome) and the allele frequencies estimated in Non-Finnish European populations in the Genome Aggregation Database (gnomAD), v. 2.1.1 [19], using a cut-off of ≥4.0 × 10^−4^ (0.04%) for germline variants [20]. Synonymous variants were filtered out and excluded from the final analysis. Variants were mapped on a linear protein and its domains using the Mutation Mapper tool and OncoPrint graph, while the distribution of genetic alterations in TET patients was generated using the OncoPrinter tool; both tools are available in cBioPortal v. 4.0.2 [21,22].

### 2.6. Statistical Analysis

Survival probabilities were calculated by the Kaplan–Meier method and differences in survival distributions were evaluated by log-rank test [23,24]. Survival analysis was performed using the MedCalc^®^ Statistical Software version 20.109 [25]. Other statistical analyses were performed using Statistica v. 10 software (StatSoft, Tulsa, OK, USA). In all analyses, the *p*-values were two-tailed and *p* < 0.05 was considered indicative of statistical significance.

## 3. Results

### 3.1. Histology and Immunohistochemical Expression of CD117

Nineteen thymomas and thirty-four thymic carcinomas were analysed; their histological subtypes are listed in Table 1. CD117 expression was observed in 30 of 33 thymic carcinomas (23 of 23 squamous cell carcinomas [SQCC], 5 of 6 large-cell neuroendocrine carcinomas [LCNEC], 1 of 1 basaloid carcinoma [BC], and 1 of 1 mucoepidermoid carcinoma [MEC]). In one case of thymic carcinoma (LCNEC), no tissue sample was available for CD117 analysis. None of the thymomas were positive for CD117. Supplementary Figures S1 and S2 show the microscopic morphology of tumours with pathogenic mutations or mutations of uncertain significance.

### 3.2. SNV Annotation and Clinical Interpretation

The samples were tested for SNVs by targeted NGS, using the TruSight Tumor 15 assay (Illumina) for variant detection in specific genomic regions (including 250 amplicons from 15 genes associated with solid tumours). The median (min–max) total read depth (coverage) was 2975 × (503–43,643) and the median (min–max) read depth of alleles that differed from the reference read (alt read depth) was 1874 × (28–39,501) for all detected SNVs. Only non-synonymous variants in the coding regions of targeted genes that potentially modulate protein structure and function, including missense, stop-gain, and frameshift changes, were analysed.

The pathogenicity of SNVs was assessed using the SIFT and PolyPhen prediction algorithms, and verified using the dbSNP, COSMIC, ClinVar, ICGC Data Portal, and VarSome databases. In total, we identified 10 pathogenic/likely pathogenic variants in four genes: *TP53* [seven variants: p.(Gly154Val), p.(Arg158Pro), p.(Leu194His), p.(Arg267ThrfsTer77), p.(Arg273Cys), p.(Arg306Ter), p.(Gln317Ter)], *ERBB2* [1 variant: p.(Val773Met)], *KIT* [one variant: p.(Leu576Pro)], and *KRAS* [one variant: p.(Gln61Leu)]. These pathogenic/likely pathogenic SNVs were heterozygous and found exclusively in thymic carcinomas. The morphologies of these tumours are shown in Supplementary Figures S1 and S2. The variants were frequently reported in databases as somatic in various solid tumour types. In the gnomAD database, most of the variants had an allele frequency of <0.00001 in Non-Finnish European populations. We also detected three variants of uncertain significance (VUS) in three genes, *ERBB2* [one variant: p.(Ser703Arg)], *KIT* [one variant: p.(Ile690Val)], and *FOXL2* [one variant: p.(Pro157Ser)], predicted to be damaging by the SIFT algorithm. All these variants were found in thymomas (B2B3, B2B3, and micronodular thymoma with lymphoid stroma, respectively; Supplementary Figure S1) and were heterozygous. In the gnomAD database, most of these variants had an allele frequency < 0.00001. The pathogenic/likely pathogenic and uncertain variants are listed in Table 2.

**Figure 1:**
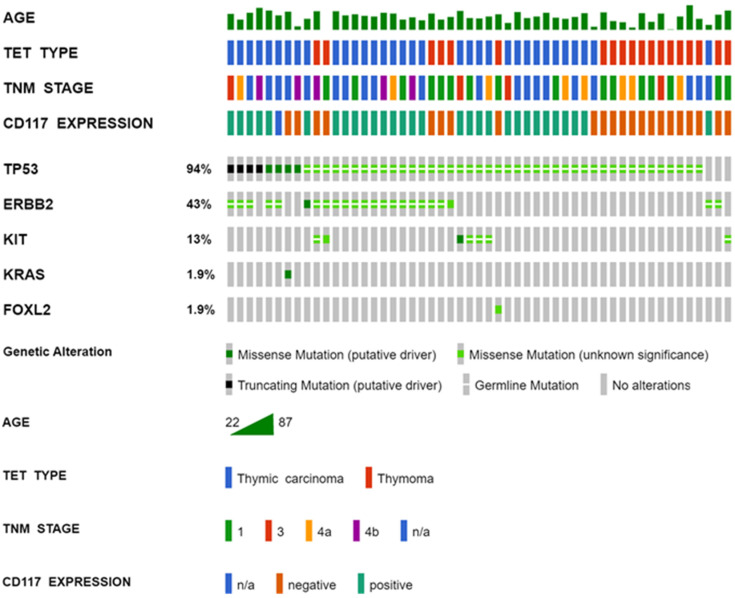
The OncoPrint showing the distribution of genetic alterations within 15 targeted genes in 53 thymic epithelial tumours (TETs). The types of mutations are labelled in the colour legend, particular genes in rows, and tumour samples in columns.

We identified several missense variants in *TP53*, *ERBB2*, and *KIT* classified as tolerated/benign by the SIFT and PolyPhen prediction algorithms and classified as benign/likely benign in clinical databases. The most frequent SNV classified as homozygous or heterozygous in 50 of 53 (94%) TET patients (33 of 34 [97%] thymic carcinomas and 17 of 19 [89%] thymomas) was the *TP53* p.(Pro72Arg) missense variant. Another frequent missense variant was *ERBB2* p.(Ile655Val), which was classified in 21 of 53 (40%) TET patients (16 of 34 [47%] thymic carcinomas and 5 of 19 [26%] thymomas) as homozygous or heterozygous. Two other recurrent SNVs were *KIT* p.(Met541Leu) (5 of 53 [9%] TET patients) and *ERBB2* p.(Ile654Val) (1 of 53 [2%] TET patients), both of which were heterozygous. In the gnomAD database, these SNVs had allele frequencies >0.04% and were thus considered germline variants. The benign variants are listed in Table 3. The distribution of pathogenic and benign variants in 15 cancer-related genes across all TETs is shown in Figure 1. The pathogenic variants and those of uncertain clinical significance are presented in the map of mutations within the linear protein sequence and its domains (Supplementary Figure S3).

### 3.3. Associations of KIT Mutations with CD117 Protein Expression

There was no correlation between *KIT* mutation status and the expression of CD117 protein. Case no. 30 (thymic carcinoma, basaloid subtype, TNM stage III), which was CD117 positive, had a pathogenic missense *KIT* p.(Leu576Pro) variant (Supplementary Figure S1B). Case no. 34 (thymic carcinoma, not otherwise specified [NOS], TNM stage not available), which was negative for CD117, had two pathogenic missense variants: *KRAS* p.(Gln61Leu) and *TP53* p.(Arg273Cys) (Supplementary Figure S1C). CD117 expression according to tumour histological type and abnormalities detected by genetic testing is shown in Figure 1 and Supplementary Table S2.

### 3.4. Associations of Pathogenic SNVs with Clinical Factors and Treatment Outcomes

**Figure 2:**
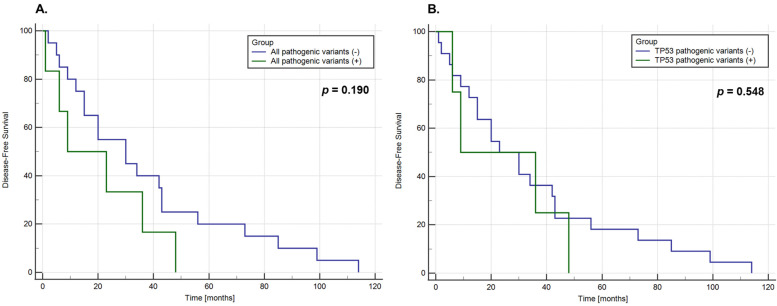
The Kaplan-Meier and Log-Rank test analysis of disease-free survival (DFS) in 34 patients with thymic carcinomas according to the presence of any pathogenic SNV found within 15 genes (**A**) or the presence of pathogenic SNV in *TP53* gene (**B**).

Because all the pathogenic variants were found in patients with thymic carcinomas, we evaluated their disease-free survival (DFS) using the Kaplan–Mayer method and log-rank test according to the presence of any pathogenic variant or a pathogenic *TP53* variant. The median (min–max) DFS in thymic carcinoma patients was 26.50 (range: 1–114) months. No significant difference in DFS was found between thymic carcinoma patients with and without pathogenic SNVs (*p* = 0.190); however, the latter showed a trend towards a longer median DFS (16.0 vs. 30.0 months, respectively; Figure 2). There was no significant difference in the median DFS between thymic carcinoma patients with and without pathogenic *TP53* variants (22.50 vs. 26.50; *p* = 0.548). The median (min–max) DFS in thymoma patients was 10 (1–87) months. No pathogenic SNV was identified in this cohort. We found no association between pathogenic SNVs and clinical factors such as age, tumour stage, and myasthenia gravis status.

## 4. Discussion

We evaluated 15 genes commonly mutated in solid tumours for non-synonymous SNVs in a retrospective series of 53 TET tissue specimens (19 [36%] thymomas and 34 [64%] thymic carcinomas) by targeted NGS. Unlike thymomas, 10 of 34 (29.4%) thymic carcinomas had at least one pathogenic variant of the analysed genes. As well as pathogenic variants known to be associated with carcinogenesis in many solid tumour types, e.g., missense, nonsense, and frameshift variants of *TP53* and activating *KRAS* variants, we found a few missense variants in *KIT* and *ERBB2* with potential as targets for small-molecule inhibitors. No pathogenic variants were found in thymomas, although a few variants of uncertain clinical significance were present in *KIT*, *ERBB2*, and *FOXL2*.

We also evaluated benign variants of possible germline origin in TETs, such as *TP53* p.(Pro72Arg) and *ERBB2* p.(Ile655Val), because they reportedly modify native-protein functions and may be risk factors for several types of solid tumour [26,27,28]. These variants were typically excluded from the analyses of prior studies.

### 4.1. Literature Review

Profiling of genetic alterations in TETs by NGS has been performed in several studies varying in terms of the total number of TETs evaluated, histological subtypes of thymomas and thymic carcinomas included, numbers of genes and amplicons sequenced, NGS approach (targeted vs. comprehensive vs. whole-exome sequencing [WES]), and sequencing platform (synthesis vs. detection of hydrogen ions). An analysis of 117 TETs by The Cancer Genome Atlas (TCGA) project using WES identified four recurrently mutated genes: general transcription factor II-I (*GTF2I*), *HRAS*, *TP53*, and *NRAS* [29]. Generally, a larger number of mutations, except in *GTF2I*, is present in thymic carcinomas compared to thymomas. *GTF2I* has a high mutation frequency (39%), particularly in type A and AB thymomas. In this study, frequently mutated genes in thymic carcinomas included *KIT*, *DDR2*, *PDGFRA*, *ROS1*, and *IGF1R*. The squamous, undifferentiated, and sarcomatoid subtypes of thymic carcinoma had the largest numbers of genomic alterations. However, the numbers of thymomas and carcinomas were imbalanced; thymomas comprising 91% of TETs.

Wang et al. performed targeted-capture sequencing of 197 cancer-related genes in a series of 47 advanced thymic carcinomas and 31 advanced thymomas [30]. Recurrent somatic non-synonymous mutations were detected in 39 genes in 33 (42%) TETs. Thymic carcinomas (29 of 47; 62%) had a higher incidence of somatic non-synonymous mutations than thymomas (4 of 31; 13%; *p* < 0.0001) and *TP53* was the most frequently mutated gene (17%), especially in thymic carcinomas (26%). Genes involved in histone modification (*BAP1*, 13%; *SETD2*, 11%; *ASXL1*, 4%), chromatin remodelling (*SMARCA4*, 4%), and DNA methylation (*DNMT3A*, 7%; *TET2*, 4%; *WT1*, 4%) were frequently mutated in thymic carcinomas, but not thymomas.

Other NGS analyses have also provided data on the mutational landscape of TETs. Enkner et al. analysed 37 thymomas and 35 thymic carcinomas by sequencing mutation hotspots in 50 genes (mostly oncogenes and tumour suppressor genes) using the Ion AmpliSeq Cancer Hotspot Panel v. 2 [31]. They detected non-synonymous mutations in *ALK*, *ATM*, *CDKN2A*, *ERBB4*, *FGFR3*, *KIT*, *NRAS*, and *TP53* in 16 (46%) thymic carcinomas. The most frequently altered gene was *TP53* (nine carcinomas). Four carcinomas exhibited a missense mutation in the tumour suppressor *CDKN2A*, which was thus the second most frequently mutated gene. Two carcinomas harboured a missense mutation in *FGFR3* or *KIT* (p.L576P, p.Y823S). Two (10%) B3 thymomas had a mutation in non-coding regions of *SMARCB1* and *STK11*. Three (17%) type A thymomas harboured a non-synonymous *HRAS* mutation.

Petrini et al. performed whole-genome sequencing (WGS) of tumour and paired normal tissues from a 55-year-old Caucasian female who underwent complete resection of a stage IVA B3 thymoma [32]. Ten SNVs in *LDB3*, *PCNXL3*, *PHF15*, *PION*, *PPP1R3A*, *SFXN3*, *SRGAP1*, *TAF1*, *VN1R5*, and *WDR70*, and two insertion/deletions (INDELs) in *FBN3* and *BCOR*, were identified; these mutations resulted in non-synonymous amino acid changes or affected splicing sites. However, none of the mutated genes were among the well-characterised cancer-associated genes described in other solid tumour types. The biological and clinical relevance of the detected variants in thymoma was unclear because of their inadequate functional characterisation.

Shitara et al. sequenced cancer-related genes in 12 thymic SQCC tissues using the Ion AmpliSeq Comprehensive Cancer Panel (all-exon coverage of 409 genes) and Ion PGM Sequencer [33]. The mutational landscape of thymic SQCC revealed by NGS analysis was highly heterogeneous. Twenty-five mutations in 24 genes were identified, including 5 tyrosine kinase genes (*KIT*, *DDR2*, *PDGFRA*, *ROS1*, *IGF1R*). However, there was no recurrent mutation among the samples studied. The *KIT* exon 11 deletion mutation (p.V556del) was found in one patient.

In three subsequent studies evaluating SNVs exclusively in thymic carcinomas, the same targeted sequencing approach based on the AmpliSeq Cancer Hotspot Panel v. 2 (Ion Torrent or Illumina platform) yielded concordant results. Asao et al. detected genetic alterations in 7 of 52 (13.5%) patients [34] in *TP53* (four patients, 7.5%), *KRAS* (two patients, 3.8%), *FBXW7* (two patients, 3.8%), and *NRAS* (one patient, 1.9%). No *KIT* mutations were detected. Sakane et al. detected 42 variants in 21 of 54 (39%) TETs, including 48 thymic carcinomas and six thymic neuroendocrine tumours. Among them, *TP53* was the most frequently mutated gene (18.5%, 12 variants), followed by *KIT* (7.4%, four variants), *PDGFRA* (5.6%, three variants), and *PIK3CA* (5.6%, three variants) [35]. There was no significant difference in mutation frequency between thymic carcinomas and thymic neuroendocrine tumours. All of the mutations in *KIT* were located in exon 11, which encodes the juxtamembrane domain, and were detected in two SQCC (variants p.E561K and p.T574del), one adenocarcinoma (p.Q575del), and one carcinoid (p.R586K). All the *KIT* variants were reported for the first time in TETs and were mutually exclusive with mutations in *PDGFRA*. Other clinically significant mutations were detected in *KRAS* (p.K16R, p.T20M), *NRAS* (p.E63K), *HRAS* (p.T20I), and *PTEN* (p.D109N). Casini et al. detected four *TP53* variants affecting the DNA binding domain (p.A276T, p.G108S, p.C275F, p.R283fs), two *KIT* variants affecting the kinase domain (p.E839K, p.R634W), a deleterious p.E17K variant in *AKT*, and a *KRAS* p.G12V variant in 10 thymic carcinomas (six epidermoid carcinomas, three undifferentiated carcinomas and one lymphoepithelioma-like carcinoma) [36]. They found no recurrent variants.

### 4.2. Pathogenic Variants

In this study, we focused on 15 genes associated with the pathogenesis of various solid tumours, including several important receptor tyrosine kinases (*EGFR*, *ERBB2*, *KIT*, *PDGFRA*, *RET*), downstream signal transducers (*AKT1*, *BRAF*, *KRAS*, *NRAS*, *PIK3CA*), a transcription factor (*FOXL2*), and a cell cycle regulator (*TP53*). Thymic carcinomas had more SNVs than thymomas. In other studies, most thymic carcinomas were of the squamous cell subtype, which showed the greatest mutational dysregulation [29,31,33,35]. We found pathogenic variants in 5 of 23 (22%) classical SqCCs (four SNVs in *TP53*, one SNV in *ERBB2*) and one BC [*KIT* p.(Leu576Pro)], which is a variant of SqCC. Among the six cases of large cell neuroendocrine carcinoma, two (33%) had pathogenic *TP53* variants [p.(Gly154Val) or p.(Gln317Ter)]. Pathogenic SNVs were also found in one adenocarcinoma (*TP53* p.(Leu194His)] and one carcinoma NOS [*TP53* p.(Arg273Cys), *KRAS* p.(Gln61Leu)]. In total, 10 of 34 (29.4%) thymic carcinomas had at least one pathogenic variant. *TP53* showed the highest frequency of pathogenic SNVs—four missense, two nonsense, and two frameshift variants in 7 of 53 (13%) TETs. This is consistent with prior reports. Finally, we detected a pathogenic *KIT* p.(Leu576Pro) variant in 1 of 34 (3%) thymic carcinomas; this frequency is similar to previous studies [30,31]. Importantly, this variant has been repeatedly reported in thymic carcinomas, mostly of the squamous cell subtype (Supplementary Table S3). This mutation is associated with low to moderate sensitivity to the tyrosine kinase inhibitors imatinib, sunitinib, dasatinib, and nilotinib in vitro [37,38]. The other pathogenic SNVs, particularly *ERBB2* p.(Val773Met) and *KRAS* p.(Gln61Leu), are still undruggable molecular targets in solid tumours, despite promising data from preclinical models, clinical trials and case reports [39,40,41,42].

### 4.3. Associations of Pathogenic Variants with DFS

There was no significant difference in DFS between thymic carcinoma patients with and without pathogenic SNVs (*p* = 0.190); however, there was a trend towards a longer median DFS in the latter group (16.0 vs. 30.0 months, respectively; Figure 2). There was no significant difference in median DFS between thymic carcinoma patients with and without pathogenic *TP53* variants (22.50 vs. 26.50 months; *p* = 0.548). Moreira et al. reported a significant difference in DFS between thymic carcinoma patients with mutated and wild-type p53 (*n* = 25, HR 0.2848, 95% CI 0.06–0.72; *p* = 0.02) [43]. The median DFS for patients with mutated p53 was 2 years (731 days), similar to our findings, but could not be determined for the p53 wild-type group. We did not evaluate p53 expression by immunohistochemistry (IHC) because the full coding sequence of *TP53* was analysed for SNVs. Xu et al. reported a significantly shorter DFS in patients with *TP53* mutated compared to wild-type *TP53* (*n* = 34, *p* = 0.003) [44]. Mutations in *TP53* are among the most frequent somatic aberrations in human cancers and are frequently reported as a negative prognostic factor for survival in many solid tumour types, including lung [45], colorectal [46], breast [47], and prostate carcinoma [48], irrespective of systemic therapy. The impact of somatic pathogenic variants on the treatment outcomes of TETs should be further evaluated in patient cohorts large enough for multivariate analysis considering histopathology, stage, and treatment regimen.

### 4.4. Variants of Uncertain Clinical Significance

In this study, 3 of 19 (16%) thymomas exhibited missense variants of uncertain clinical significance in *ERBB2* [p.(Ser703Arg)], *KIT* [p.(Ile690Val)], and *FOXL2* [p.(Pro157Ser)]. The *ERBB2* and *KIT* variants were detected in B2B3 thymomas, and the *FOXL2* variant in a micronodular thymoma with lymphoid stroma. Missense variants in *ERBB* family genes have only occasionally been reported in thymomas [30,49,50]. The missense *ERBB2* p.(Ser703Arg) variant has not been referred to elsewhere; there is no information on its clinical significance in genetic databases and no entry in gnomAD. This variant was heterozygous and had an allele frequency of 10% (888 total reads), suggesting a somatic origin. In tumour genomic profiling, pathogenic variants are assumed to be of germline origin when the allele frequency is approximately 50%, and a value of 30–70% is accepted as representative of a heterozygous pathogenic variant [51,52,53]. The *ERBB2* p.(Ser703Arg) variant in exon 18 causes an amino acid change from serine to arginine at position 703 in the juxtamembrane domain of HER2 (Supplementary Figure S3) [54]. Recent research showed evidence that apart from well-studied oncogenic mutations in the extracellular and kinase domains of HER2, mutations in transmembrane and juxtamembrane domains may also activate signalling by enhancing the active dimer interface or stabilising the activating conformation. In gastric carcinoma, *ERBB2* mutations in the juxtamembrane domain showed the highest frequency and were not associated with HER2 immunohistochemical overexpression [55]. Likewise, in thymomas, HER2/neu protein overexpression is infrequent [31,56,57,58].

*KIT* p.(Ile690Val) was found in one thymoma. To our knowledge, this variant has not been reported in TETs in either somatic or germline form (Supplementary Table S2). In our analysis, this variant was heterozygous and had an allele frequency of 54% (684 total reads), suggesting a germline origin. On the one hand, this sequence change replaces isoleucine with valine at codon 690 in the kinase insert domain of the KIT protein (Supplementary Figure S3) [59]. On the other hand, the isoleucine residue is highly conserved and there is a small physicochemical difference between isoleucine and valine. Algorithms developed to predict the effects of missense changes on protein structure and function (e.g., SIFT, PolyPhen) indicated that this variant is likely to be tolerated, but this has not been confirmed by functional studies and the clinical significance is uncertain. Therefore, scientific evidence for the mutational activation of *KIT* p.(Ile690Val) is lacking. This variant has two entries (both germline variants) in the ClinVar database: gastrointestinal stromal tumour (GIST; accession RCV000538990.4) and hereditary cancer-predisposing syndrome (RCV001014301.1). Because of the lack of supporting evidence, the clinical significance of this alteration is unclear.

The third VUS, *FOXL2* p.(Pro157Ser), is more enigmatic than the two mentioned above. Alterations in *FOXL2* have not previously been associated with TETs. However, *FOXL2* was not analysed in most studies of genetic alterations in thymomas or thymic carcinomas by targeted sequencing because it is absent from NGS panels [31,44,49,50,60]. In our analysis, this variant was heterozygous and had an allele frequency of 6% (4905 total reads), suggesting a somatic origin. This variant has one entry in the cBioPortal for Cancer Genomics for two colon adenocarcinomas (samples P-0023271-T01-IM6 and P-0018374-T01-IM6) at frequencies <1% in the MSK MetTropism cohort [61]. FOXL2 is a transcription factor of the forkhead box (FOX) superfamily and is implicated in the development and functioning of the ovaries [62]. Mutations in *FOXL2* are mostly found in ovarian granulosa cell tumours [63,64]. The other transcription factor, general transcription factor II-I (TFII-I), is a multifunctional phosphoprotein critical for transcription and signal transduction. It is encoded by *GTF2I*, which has a high frequency of a missense mutation (chromosome 7 c.74146970T>A), particularly in type A thymomas (followed by type AB and type B thymomas) [29,65,66]. Because thymomas show molecular dysregulation in terms of transcription factors, we consider *FOXL2* to be a molecular target meriting further investigation.

Unlike other authors, we did not identify pathogenic variants of *TP53*, *KRAS*, *PIK3CA*, *AKT1*, *EGFR*, or *RET* in thymomas [30,35,38,49,50,67]. Significant enrichment of mutated genes of the RAS and PI3K-Akt signalling pathways was reported in thymomas (*AKT3*, *ALK*, *CSF1R*, *FGFR4*, *KRAS*, *NRAS*, *HRAS*, *PIK3CA*), and their role in thymoma development was suggested [30,31,35,38,44,49].

### 4.5. Germline Variants

None of the referenced studies of genetic alterations in TETs reported germline non-synonymous SNVs. We identified several germline missense variants in *TP53*, *ERBB2*, and *KIT*. The most frequent SNV (94% of TETs) was the *TP53* p.(Pro72Arg) missense variant. A common single nucleotide polymorphism (SNP) at codon 72 of p53 is one of the most widely studied variations in *TP53* [26]. The codon 72 SNP is in exon 4, in a segment of *TP53* encoding the proline-rich domain (PRD) of p53, which is crucial for apoptosis induction in response to stress [68,69,70,71]. The data suggest that the Pro72 and Arg72 variants differentially modify the function of p53 based on their altered transcriptional and apoptotic potential. This is the result of an altered ability to bind to p53 target promoters and transcriptional regulators [26]. Genome-wide association studies have failed to identify the codon 72 SNP as a risk factor for cancer, because such studies are unable to control for factors interacting with the SNP and cancer risk, such as environmental factors and mutation of specific driver oncogenes in a particular tumour type [72]. However, the codon 72 SNP reportedly modulates the ability of mutant p53 protein to perform its gain-of-function activities in tumours with pathogenic *TP53* variants [73,74].

The *ERBB2* p.(Ile655Val) variant was detected in 40% of the tumours in this study. This is the most extensively investigated *ERBB2* polymorphism related to cancer risk and is located at codon 655 (ATC/isoleucine to GTC/valine) in the transmembrane domain of the HER2 receptor (exon 17) [75]. Such an amino acid change could increase tyrosine kinase activity. Ile655Val is present in several malignancies, including breast, gastric, colorectal, and lung carcinomas [75,76,77,78]. Tumours harbouring the Ile655Val polymorphism exhibited higher tumourigenic potential in preclinical models and an aggressive phenotype [79]. In one SQCC, we identified two concomitant *ERBB2* variants: p.(Ile654Val) and p.(Ile655Val). Val654 is reportedly linked to Val655, resulting in two consecutive valines instead of two isoleucines in the transmembrane domain. Ile654 and Ile655 are highly conserved among species [80]. By analysing the transmembrane segments of an ERBB2 homodimer, Fleishman et al. characterised two stable conformations of the transmembrane domain [27]. Based on this molecular switch model for the activation of ERBB2, it is hypothesised that substitution of Ile654 for valine stabilises the formation of active ERBB2 dimers, thus increasing autophosphorylation, tyrosine kinase activity, and cell transformation. Therefore, by exchanging these two consecutive isoleucines for two valines, the effect on active dimer formation is enhanced [80].

In this study, the germline *KIT* p.(Met541Leu) variant was found in 9% of TETs. To date, Met541Leu has mostly been reported in GISTs, in which it is associated with an increased risk of tumour progression and dissemination [81,82,83]. Using transfected cell lines, Brahmi et al. found that *KIT* p.(Met541Leu) induced mutant-like intracellular signalling in the presence of stem cell factor [81]. In vitro, cells expressing Met541Leu had a proliferative and survival advantage. The substitution of methionine for leucine at position 541, at the end of the transmembrane domain (exon 10), increased activation by the KIT receptor of the PI3K/AKT and MAPK signalling pathways compared to wild-type KIT. However, *KIT* p.(Met541Leu) was not associated with responses to imatinib in GISTs [81,83], aggressive fibromatosis [84], or hypereosinophilia [85].

### 4.6. Limitations

This study had several limitations. The total number of 53 analysed TETs may seem to be small, but this is comparable with similar prior studies [34,44,49,60,67,86]. It should be emphasized, however, that the tumour specimens investigated were collected at the two clinical centres; TETs are rare tumours (incidence about 0.13 per 100,000 person-years) [87]. The limited number of TETs hampered survival analysis—DFS differed between groups, but not significantly. We did not evaluate the overall survival of TET patients because several cases were lost to follow-up, while in others the clinical endpoint was not achieved. Therefore, multivariate analysis of survival in a larger patient cohort is needed. Finally, the molecular analysis was restricted to SNVs in 15 genes; however, these included genes commonly mutated in solid tumours that regulate important signalling pathways (e.g., the p53, AKT, MAPK, PIK3CA, and Ras pathways), and are implicated in carcinogenesis. We achieved a high total read depth (coverage) for the detected SNVs and used stringent quality criteria to avoid common sequencing artifacts, thus enhancing the reliability of the results.

## 5. Conclusions

Thymic carcinomas showed greater genetic dysregulation than thymomas, in terms of SNVs in the coding sequences of 15 genes implicated in the pathogenesis of solid tumours. Ten of thirty-four (29.4%) thymic carcinomas had at least one pathogenic/likely pathogenic variant considered to be somatic. Some of those variants, such as *ERBB2* p.(Val773Met), have not been reported in TETs. Importantly, druggable KIT alterations in thymic carcinomas, such as *KIT* p.(Leu576Pro), have potential as therapeutic targets. Several clinical trials (e.g., NCT02220855, NCT02049047, NCT00369889, and NCT03449173) have evaluated, or are evaluating, the clinical efficacy of molecularly targeted drugs in TET patients with genetic alterations of predictive value. Precision medicine for TETs necessitates identification of druggable molecular targets in these rare and challenging tumours. No somatic SNVs confirmed as pathogenic/likely pathogenic were found in the 19 thymomas analysed in this study.

It is important to report germline and rare SNVs of uncertain clinical significance, such as alterations in *FOXL2*, to add to the number of known genetic alterations in TETs and provide molecular insight into these neoplasms. Our findings suggest the need for comprehensive genomic profiling of TETs.

## Figures and Tables

**Table 1:** Clinicopathological characteristics of 53 patients with thymic epithelial tumours.

Characteristics	Thymomas	N (%)	Thymic Carcinomas *	N (%)	Total
Number of cases	All	19 (36%)	All	34 (64%)	53 (100%)
Median age (range) in years		57 (22–87)		57 (30–80)	57 (22–87)
Gender	Male	13 (25%)	Male	24 (45%)	37 (70%)
	Female	6 (11%)	Female	10 (19%)	16 (30%)
Histology (WHO 2021)	A	1 (2%)	Squamous cell carcinoma **	23 (43%)	
	AB	4 (8%)	Basaloid carcinoma	1 (2%)	
	B2	5 (9%)	Adenocarcinoma	1 (2%)	
	B3	1 (2%)	Mucoepidermoid carcinoma	1 (2%)	
	Combined B2B3	5 (9%)	NUT carcinoma	1 (2%)	
	MTLS	3 (6%)	LCNEC	6 (11%)	
			Carcinoma, NOS	1 (2%)	
Stage (TNM)	I	12 (23%)	I	4 (8%)	
	II	0 (0%)	II	0 (0%)	
	III (A + B)	1 (2%)	III (A + B)	3 (6%)	
	IV (A + B)	4 (8%)	IV (A + B)	9 (17%)	
	*n*/*a*	2 (4%)	*n*/*a*	18 (34%)	
Myasthenia gravis	Yes	5 (9%)	Yes	0 (0%)	
	No	14 (26%)	No	34 (64%)	
Adjuvant treatment	None	7 (13%)	None	2 (4%)	
	RTH	8 (15%)	RTH	5 (9%)	
	CHTH	1 (2%)	CHTH	5 (9%)	
	RTH + CHTH	2 (4%)	RTH + CHTH	13 (25%)	
	*n*/*a*	1 (2%)	*n*/*a*	9 (17%)	

MTLS = Micronodular thymoma with lymphoid stroma; NUT = Nuclear protein in testis; LCNEC = Large cell neuroendocrine carcinoma; NOS = Not otherwise specified; RTH = Radiotherapy; CHTH = Chemotherapy; *n*/*a* = data not available. * Including large cell neuroendocrine carcinomas; ** Including micronodular carcinoma with lymphoid hyperplasia.

**Table 2:** The summary of single nucleotide variants, which clinical significance is considered as pathogenic/likely pathogenic or uncertain (VUS), identified in thymic epithelial tumours.

Sample ID	TET Histology	TET Subtype	Gene	HGVSC	HGVSP	Variant Position	Allele Frequency (GnomAD)	dbSNP	COSMIC ID	Mutation Type	Clinical Significance (VarSome)	Variant Read Frequency	Coverage [Reads]
#29	TC	LNEC	*TP53*	c.461G>T	p.(Gly154Val)	chr17:7578469	*n*/*a*	rs762846821	COSM6815	missense	likely pathogenic	0.82	3107
#19	TC	SQCC	*TP53*	c.473G>C	p.(Arg158Pro)	chr17:7578457	*n*/*a*	rs587782144	COSM43615	missense	pathogenic	0.45	4832
#31	TC	ADC	*TP53*	c.581T>A	p.(Leu194His)	chr17:7578268	0.000	rs1057519998	COSM43623	missense	pathogenic	0.21	4354
#8	TC	SQCC	*TP53*	c.799_802del	p.(Arg267ThrfsTer77)	chr17:7577136	*n*/*a*	*n*/*a*	*n*/*a*	frameshift	pathogenic	0.80	2506
#34	TC	NOS	*TP53*	c.817C>T	p.(Arg273Cys)	chr17:7577121	0.000	rs121913343	COSM10659	missense	pathogenic	0.73	1929
#3	TC	SQCC	*TP53*	c.916C>T	p.(Arg306Ter)	chr17:7577022	0.000	rs121913344	COSM10663	stop gain	pathogenic	0.44	3338
#5	TC	SQCC	*TP53*	c.916C>T	p.(Arg306Ter)	chr17:7577022	0.000	rs121913344	COSM10663	stop gain	pathogenic	0.38	2395
#26	TC	LNEC	*TP53*	c.949C>T	p.(Gln317Ter)	chr17:7576897	*n*/*a*	rs764735889	COSM10786	stop gain	pathogenic	0.90	19,414
#50	TM	B2B3	*ERBB2*	c.2109C>G	p.(Ser703Arg)	chr17:37879814	*n*/*a*	*n*/*a*	*n*/*a*	missense	uncertain	0.10	888
#18	TC	SQCC	*ERBB2*	c.2317G>A	p.(Val773Met)	chr17:37880988	0.000015	rs772054394	COSM5731177	missense	pathogenic	0.07	2483
#30	TC	Basaloid	*KIT*	c.1727T>C	p.(Leu576Pro)	chr4:55593661	*n*/*a*	rs121913513	COSM1290	missense	pathogenic	0.41	740
#49	TM	B2B3	*KIT*	c.2068A>G	p.(Ile690Val)	chr4:55595578	0.000009	rs924104591	*n*/*a*	missense	uncertain	0.53	684
#34	TC	NOS	*KRAS*	c.182A>T	p.(Gln61Leu)	chr12:25380276	0.000	rs121913240	COSM553	missense	pathogenic	0.87	38,464
#52	TM	MTLS	*FOXL2*	c.469C>T	p.(Pro157Ser)	chr3:138665096	0.000	rs758370933	*n*/*a*	missense	uncertain	0.06	4905

TM = thymoma; TC = Thymic carcinoma; SQCC = Squamous cell carcinoma; ADC = Adenocarcinoma; LCNEC = Large cell neuroendocrine carcinoma; MTLS = Micronodular thymoma with lymphoid stroma; NOS = Not otherwise specified; *n*/*a* = data not available. *TP53*—Tumour protein p53, *ERBB2*—Erb-B2 Receptor Tyrosine Kinase; *KIT*—KIT Proto-Oncogene, Receptor Tyrosine Kinase; *KRAS*—Protein V-Ki-ras2 Kirsten rat sarcoma viral oncogene homolog; *FOXL2*-Forkhead Box L2. Reference transcripts: NM_000546.6 for *TP53*, NM_004448.3 for *ERBB2*, NM_000222.2 for *KIT*, NM_033360.3 for *KRAS*, NM_023067.3 for *FOXL2* gene.

**Table 3:** The summary of single nucleotide variants, which clinical significance is considered as benign, identified in thymic epithelial tumours.

Gene	HGVSC	HGVSP	Variant Position	Variant Frequency in TETs	Allele Frequency (GnomAD)	dbSNP	COSMIC ID	Mutation Type	Clinical Significance (VarSome)	Variant Read Frequency	Median Coverage [Reads]
*TP53*	c.215C>G	p.(Pro72Arg)	chr17:7579472	50/53 (94%)	0.7366	rs1042522	COSM250061	missense	benign	0.19–1.00	17,854
*ERBB2*	c.1963A>G	p.(Ile655Val)	chr17:37879588	21/53 (40%)	0.2405	rs1136201	COSM4000121	missense	benign	0.23–1.00	2600
*ERBB2*	c.1960A>G	p.(Ile654Val)	chr17:37879585	1/53 (2%)	0.0081	rs1801201	COSM6854579	missense	benign	0.59	2718
*KIT*	c.1621A>C	p.(Met541Leu)	chr4:55593464	5/53 (9%)	0.0969	rs3822214	COSM28026	missense	benign	0.45–0.53	2198

*TP53*—Tumour protein p53, *ERBB2*—Erb-B2 Receptor Tyrosine Kinase; *KIT*—KIT Proto-Oncogene, Receptor Tyrosine Kinase. Reference transcripts: NM_000546.6 for *TP53*, NM_004448.3 for *ERBB2*, NM_000222.2 for *KIT* gene.

## Data Availability

The detailed data presented in this study are available from the corresponding authors on reasonable request.

## Supplementary Materials

The following supporting information can be downloaded at: https://www.mdpi.com/article/10.3390/cancers14143388/s1, Figure S1: Microscopic morphology of tumours presenting pathogenic mutations or mutations of uncertain significance in genes other than *TP53*. Table S1: The list of 15 genes and their regions covered by the TruSight Tumor 15 sequencing panel. Figure S2: Microscopic morphology of tumours presenting pathogenic mutations in *TP53* gene. Table S2: The summary results of single nucleotide variants (SNV) analysis by NGS and KIT (CD117) expression evaluation by IHC in thymic carcinomas and thymomas. Figure S3: The scheme of protein sequence and functional domains of *TP53*, *ERBB2*, *KIT*, and *KRAS* genes with the locations of amino acids changes introduced by missense (green pin) and nonsense single nucleotide variants, SNVs (black pin). Table S3: The summary of a literature data on the type and frequency of *KIT* mutations in patients with thymic carcinoma and preclinical or clinical efficacy of KIT tyrosine kinase inhibitors [30,31,33,35,36,38,67,86,88,89,90,91,92,93,94,95,96,97].
