# Supplementary material for: Targeted Next-Generation Sequencing of Thymic Epithelial Tumours Revealed Pathogenic Variants in KIT, ERBB2, KRAS, and TP53 in 30% of Thymic Carcinomas
Source: Cancers (Basel). 2022 Jul 12;14(14):3388. doi: 10.3390/cancers14143388 (PMC9324890; doi:10.3390/cancers14143388)
Supplement: Supplementary file 1 [file cancers-14-03388-s001.zip › Szpechcinski_Szolkowska - NGS analysis of 53 thymic epithelial tumors - Figure S1.pdf]

SUPPLEMENTARY MATERIAL

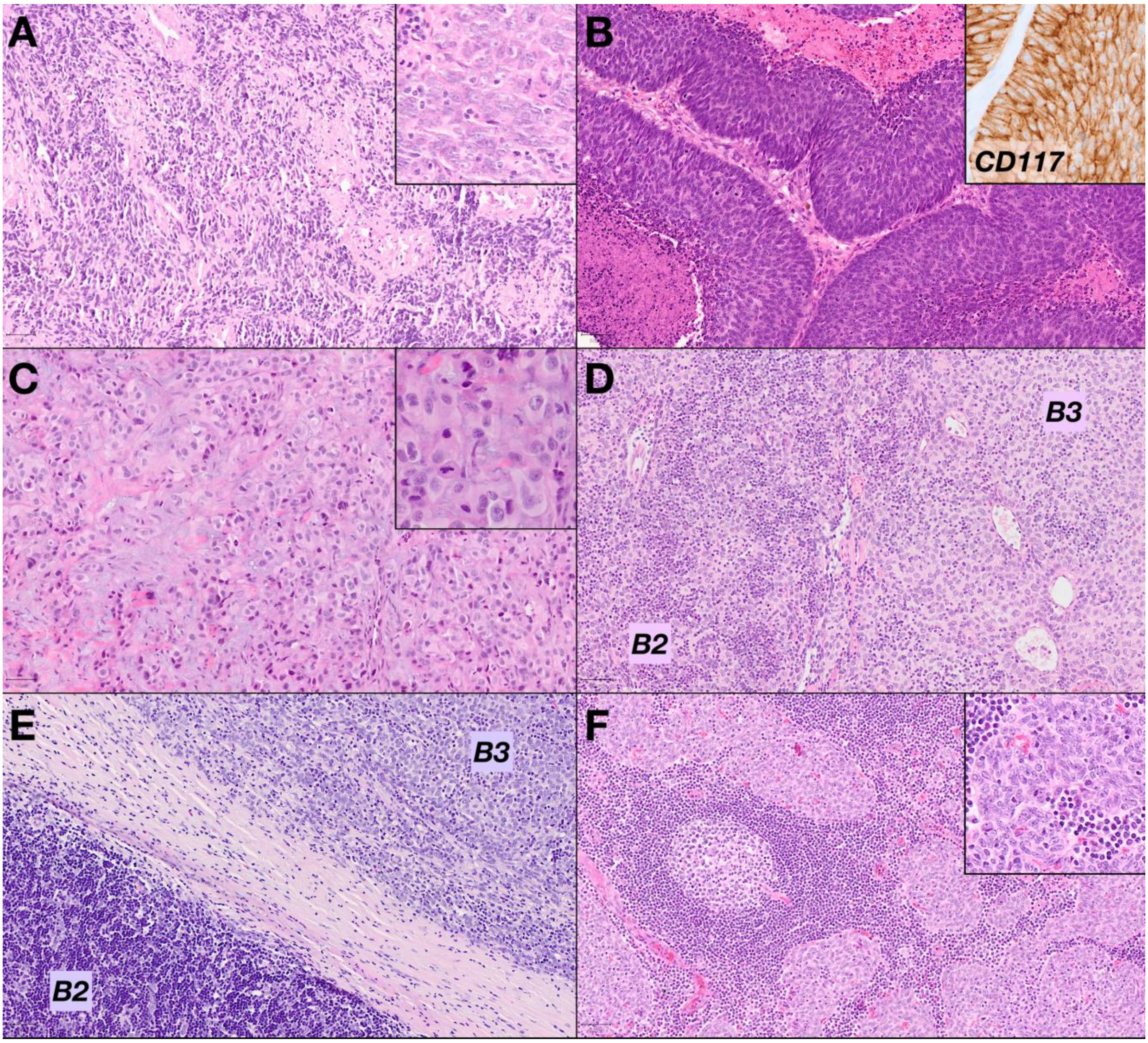

Figure S1. Histopathology of tumours with pathogenic mutations or mutations of uncertain significance in genes other than *TP53* (SqCC = Squamous cell carcinoma, IHC = immunohistochemistry, NGS = next generation sequencing).

A. Case no. 18: Thymic SqCC, TNM stage not available, 52-year-old woman. Under low magnification, a monotonous population of poorly differentiated tumour cells that do not form any organoid structures was visible. Under higher magnification (inset) the cells were large with vesicular, irregular cell nuclei and distinct, eosinophilic nucleoli. IHC: positive for AE1AE3, p63, CK5/6 (+ in singular cells), CD117 and CD5 and negative for TTF-1. NGS: pathogenic mutation in *ERBB2* gene [p.(Val773Met)];

B. Case no. 30: Thymic basaloid carcinoma, TNM stage III, 73-year-old man. Carcinoma nests are formed by densely packed, multilayered neoplastic cells with peripheral palisading and comedo-like necrosis (lower, left). IHC: positive for p63, CD5, CD117 (inset) and negative for CD56, synaptophysin and TTF-1. NGS: pathogenic mutation in *KIT* gene [p.(Leu576Pro)].

C. Case no. 34: Thymic carcinoma, not otherwise specified (NOS), TNM stage not available, 70-year-old man. The tumour was composed of large epithelioid cells with abundant cytoplasm, irregular cell nuclei and distinct nucleoli. IHC: positive for AE1AE3, EMA, CK5/6 (in some cells), BerEp4 and negative for p40, TTF-1, CEA, calretinin, WT-1, CD5 and CD117. Mucin: negative. The point of origin

(i.e. thymus) was confirmed by radiological findings. NGS: pathogenic mutation in *KRAS* [p.(Gln61Leu)] and *TP53* [p.(Arg273Cys)] genes.

D. Case no. 49: Type B2B3 thymoma, TNM stage I, 23-year-old man with myasthenia gravis. Two intermingled components were seen - lymphocyte-rich one with large epithelial cells loosely dispersed or forming small groups among lymphocytes. Morphology of this component corresponded with type B2 thymoma (*B2*). The second component, corresponding with type B3 thymoma, was lymphocyte-poor and built with sheets of epithelial cells with palisading around small vessels (*B3*). IHC: positive for AE1AE3, EMA (focally) and D2-40. NGS: mutation of uncertain significance in *KIT* gene [p.(Ile690Val)].

E. Case no. 50: Type B2B3 thymoma, TNM stage I, 56-year-old man with myasthenia gravis. Morphologically similar tumour to the previous case. The two visible components presenting type B2 and B3 features are separated by the fibrous interlobular septum. IHC: not performed. NGS: mutation of uncertain significance in *ERBB2* gene [p.(Ser703Arg)].

F. Case no. 52: Micronodular thymoma with lymphoid stroma, TNM stage I, 63-year-old woman. The tumour was composed of confluent nodules formed by spindle epithelial cells (inset). The stroma was rich in B lymphocytes with lymphoid follicles containing distinct germinal centres (left). IHC: not performed. NGS: mutation of uncertain significance in *FOXL2* gene [p.(Pro157Ser)].

[Hematoxylin and eosin stain, magnification x100 and x200 (insets)].
