# Supplementary material for: Targeted Next-Generation Sequencing of Thymic Epithelial Tumours Revealed Pathogenic Variants in KIT, ERBB2, KRAS, and TP53 in 30% of Thymic Carcinomas
Source: Cancers (Basel). 2022 Jul 12;14(14):3388. doi: 10.3390/cancers14143388 (PMC9324890; doi:10.3390/cancers14143388)
Supplement: Supplementary file 1 [file cancers-14-03388-s001.zip › Szpechcinski_Szolkowska - NGS analysis of 53 thymic epithelial tumors - Figure S2.pdf]

SUPPLEMENTARY MATERIAL

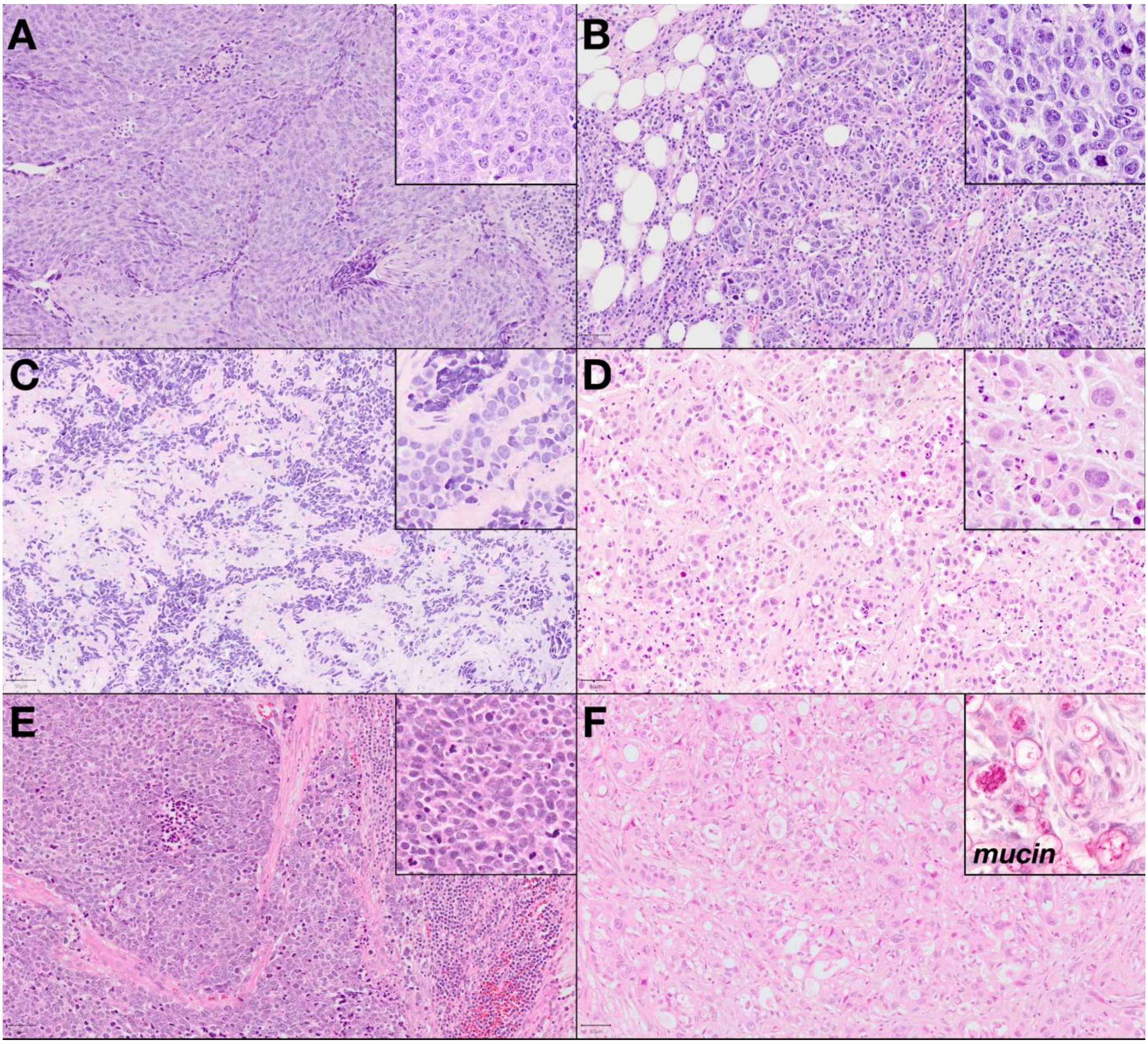

Figure S2. Histopathology of tumours with pathogenic mutations in *TP53* gene (SqCC = Squamous cell carcinoma, LCNEC = Large cell neuroendocrine carcinoma, IHC = immunohistochemistry, NGS = next generation sequencing). Microscopic slides of case no. 29 were not available at the time the photos were taken for publication.

A. Case no. 3: Thymic SqCC, TNM stage III, 65-year-old man. Solid tumour composed of nests of neoplastic cells containing large, vesicular nuclei and distinct nucleoli (inset). IHC: positive for CD5, CD117 and p63, negative for TTF-1. NGS: mutation in *TP53* gene [p.(Arg306Ter)].

B. Case no. 5: Thymic SqCC, TNM stage IVa, 50-year-old woman. Microscopically poorly differentiated non-small cell carcinoma with focal squamous cell differentiation (not shown) and high mitotic index (inset) was found. Infiltration of adipose tissue could be appreciated. IHC: positive for p40 (focally), CD5 and CD117. NGS: mutation in *TP53* gene [p.(Arg306Ter)]

C. Case no. 8: Thymic SqCC, TNM stage not available, 68-year-old woman. Another case of poorly differentatied carcinoma. Nests of neoplastic cells were embedded in desmoplastic stroma. IHC: positive for AE1AE3, p40 and CD117, negative for CD5, TTF-1 (-), CD56/NCAM, LCA, CD3 and CD20. NGS: mutation in *TP53* gene [p.(Arg267ThrfsTer77)].

D. Case no. 19: Thymic SqCC, TNM stage not available, 73-year-old woman. A tumour was composed of large cells with abundant cytoplasm. At higher magnification a slight pleomorphism of neoplastic cells was noticeable (inset). IHC: positive for p63, CD5 (focally), CD117 (weak), negative for TTF-1. NGS: mutation in *TP53* gene [p.(Arg158Pro)].

E. Case no. 26: Thymic LCNEC, TNM stage IVb, 80-year-old woman. Microscopically solid tumour composed of monotonous population of poorly differentiated neoplastic cells (inset) and foci of comedo-like necrosis (left) was seen. Lymphoid stroma on the right represented the remnants of the thymic gland. The patient had concomitant well-differentiated squamous cell carcinoma of the lung (not shown). IHC: positive for AE1/AE3, CD117, CD5 (focally), CD56/NCAM, chromogranin A and synaptophysin (focally), negative for p40. NGS: mutation in *TP53* gene [p.(Gln317Ter)].

F. Case no. 31: Thymic adenocarcinoma, TNM stage IVb, 31-year-old-man. Neoplastic cells were large with abundant cytoplasm and irregular, vesicular nuclei. Multiple microcysts containing mucin-positive substance (inset) were found within the tumour structure. IHC: positive for CK5/6 (weak and focal), negative for p63, CD5, CD117, TTF-1. Histochemical stain for mucin (mucicarmine) positive. NGS: mutation in *TP53* gene [p.(Leu194His)].

[Hematoxylin and eosin stain, magnification x100 and x200 (insets)].
