# Supplementary material for: Targeted Next-Generation Sequencing of Thymic Epithelial Tumours Revealed Pathogenic Variants in KIT, ERBB2, KRAS, and TP53 in 30% of Thymic Carcinomas
Source: Cancers (Basel). 2022 Jul 12;14(14):3388. doi: 10.3390/cancers14143388 (PMC9324890; doi:10.3390/cancers14143388)
Supplement: Supplementary file 1 [file cancers-14-03388-s001.zip › Szpechcinski_Szolkowska - NGS analysis of 53 thymic epithelial tumors - Figure S3.pdf]

SUPPLEMENTARY MATERIAL

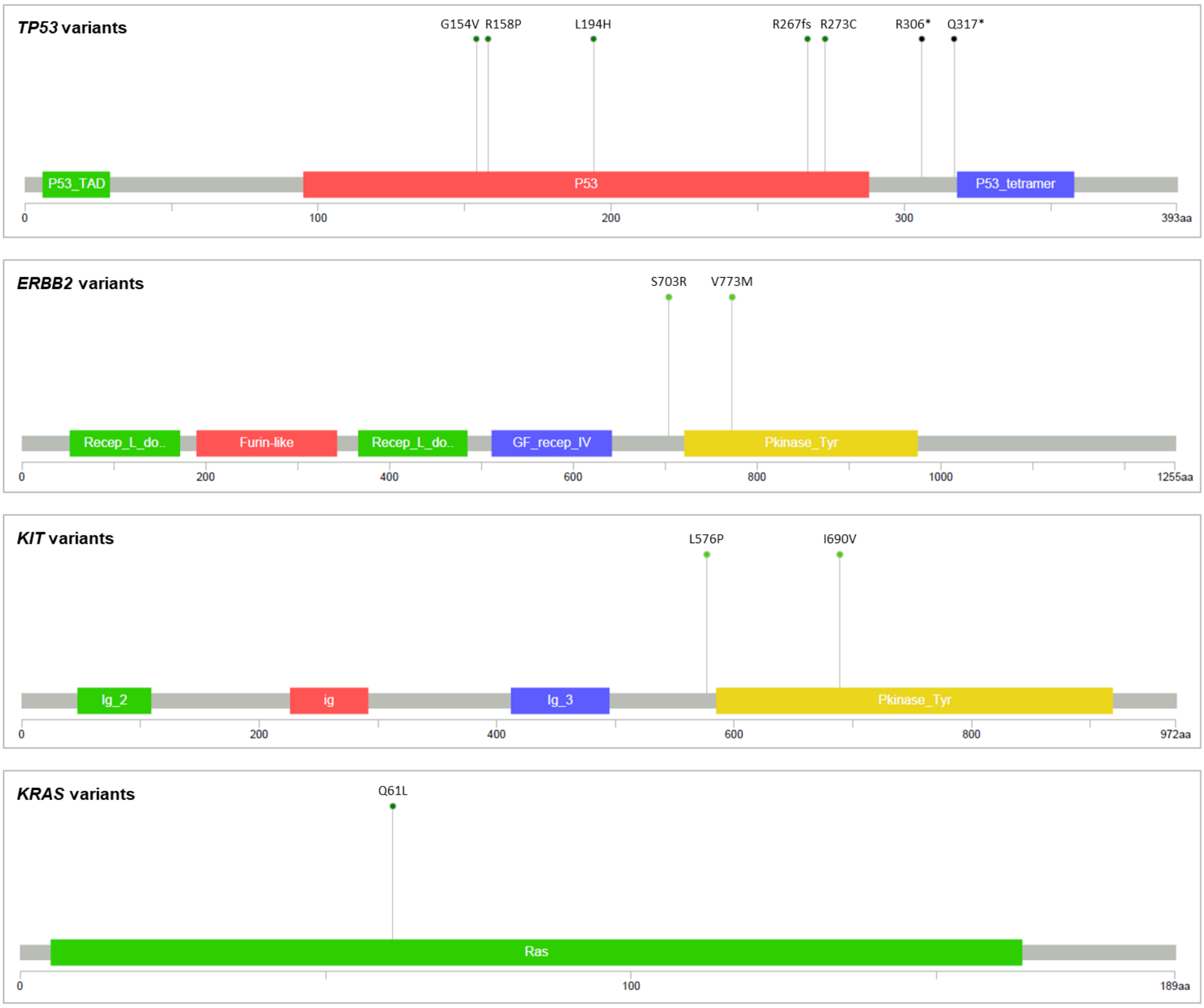

Figure S3. The scheme of protein sequence and functional domains of *TP53*, *ERBB2*, *KIT*, and *KRAS* genes with the locations of amino acids changes introduced by missense (green pin) and nonsense single nucleotide variants, SNVs (black pin). Only pathogenic/likely pathogenic SNVs and variants of uncertain clinical significance (VUS) found in 53 TETs were shown.
