# Supplementary material for: Targeted Next-Generation Sequencing of Thymic Epithelial Tumours Revealed Pathogenic Variants in KIT, ERBB2, KRAS, and TP53 in 30% of Thymic Carcinomas
Source: Cancers (Basel). 2022 Jul 12;14(14):3388. doi: 10.3390/cancers14143388 (PMC9324890; doi:10.3390/cancers14143388)
Supplement: Supplementary file 1 [file cancers-14-03388-s001.zip › Szpechcinski_Szolkowska - NGS analysis of 53 thymic epithelial tumors - Table S1.pdf]

SUPPLEMENTARY MATERIAL

Table S1. The list of the 15 genes and their regions covered by the TruSight Tumor 15 sequencing panel.

AKT1	Exon 3* ; E17K	***GNA11***	Exon 5*; Q209L	***NRAS***	Exons 2* , 3* (partial), 4 Codons 12, 13, 59, 61, 117, 146
BRAF	Exon 15* (partial); V600E/K/R/M	***GNAQ***	Exon 5* (partial); Q209L	***PDGFRA***	Exons 12, 14, 18
EGFR	Focal Amplification, Exons 12* (partial), 18, 19, 20; G719A, G719X; Exon 21 (L858R), L861Q, S7681, T790M	***KIT***	Exons 8, 9, 10, 11, 13, 14, 17, 18	***PIK3CA***	Exons 9, 20
ERBB2	Focal Amplification, p.E770_A771insAYVM Exons 14* (partial), 17, 18, 19, 20* (partial), 21* (partial), 24, 26	***KRAS***	Exon 2* (partial), 3* (partial), 4	***RET***	Exon 16 (M918T)
FOXL2	Exon 1* (partial); C134W	***MET***	Focal Amplification	***TP53***	Full coding sequence

Genes: *AKT1* – AKT Serine/Threonine Kinase 1; *BRAF* – B-Raf Proto-Oncogene, Serine/Threonine Kinase; *EGFR* – Epidermal Growth Factor Receptor; *ERBB2* – Erb-B2 Receptor Tyrosine Kinase; *FOXL2* – Forkhead Box L2; *GNA11* – G Protein Subunit Alpha 11; *GNAQ* – G Protein Subunit Alpha Q; *KIT* – KIT Proto-Oncogene, Receptor Tyrosine Kinase; *KRAS* – Protein V-Ki-ras2 Kirsten rat sarcoma viral oncogene homolog; *MET* - MET Proto-Oncogene, Receptor Tyrosine Kinase; *NRAS* – NRAS Proto-Oncogene, GTPase; *PDGFRA* – Platelet Derived Growth Factor Receptor Alpha; *PIK3CA* – Phosphatidylinositol-4,5-Bisphosphate 3-Kinase Catalytic Subunit Alpha; *RET* – Ret Proto-Oncogene; *TP53* – Tumour protein P53. *Coverage of these exons is only partial and targets specific hotspot.
