# Supplementary material for: Targeted Next-Generation Sequencing of Thymic Epithelial Tumours Revealed Pathogenic Variants in KIT, ERBB2, KRAS, and TP53 in 30% of Thymic Carcinomas
Source: Cancers (Basel). 2022 Jul 12;14(14):3388. doi: 10.3390/cancers14143388 (PMC9324890; doi:10.3390/cancers14143388)
Supplement: Supplementary file 1 [file cancers-14-03388-s001.zip › Szpechcinski_Szolkowska - NGS analysis of 53 thymic epithelial tumors - Table S2.pdf]

SUPPLEMENTARY MATERIAL

Table S2. The summary results of single nucleotide variant (SNV) analysis by NGS and KIT (CD117) expression evaluation by IHC in thymomas and thymic carcinomas. The SNVs were annotated as the protein sequence name (HGVSP) according to recommendations of Human Genome Variation Society.

Patient No.		1	2	3	4	5	6	7	8	9	10	11	12	13	14	15	16	17	18	19	20	21	22	23	24	25	26	27	28	29
Stage		n/a	n/a	3	3	4b	n/a	n/a	n/a	2a	n/a	n/a	n/a	4b	3	3	5b	n/a	n/a	n/a	n/a	2b	2b	4b	4a	n/a	4b	4b	n/a	3
Myasthenia gravis		-	-	-	-	-	-	-	-	-	-	-	-	-	-	-	-	-	-	-	-	-	-	-	-	-	-	-	-	-
TET histology		THYMIC CARCINOMA																												
Gene	Variant	SQCC																							LNEC					
TP53	P72R	● (blue)		● (blue)	● (blue)	● (blue)	● (blue)	● (blue)	● (blue)	● (blue)	● (blue)	● (blue)	● (blue)	● (blue)	● (blue)	● (blue)	● (blue)	● (blue)	● (blue)	● (blue)	● (blue)	● (blue)	● (blue)	● (blue)	● (blue)	● (blue)	● (blue)	● (blue)	● (blue)	● (blue)
	G154V																													● (red)
	R158P																			● (red)										
	L194H																													
	R267fs								► (red)																					
	R273C																													
	R306*			▼ (red)		▼ (red)																								
	Q317*																										▼ (red)			
ERBB2	I654V										● (blue)																			
	I655V	● (blue)	● (blue)	● (blue)		● (blue)		● (blue)	● (blue)	● (blue)	● (blue)	● (blue)		● (blue)			● (blue)			● (blue)		● (blue)		● (blue)		● (blue)				● (blue)
	S703R																													
	V773M																		● (red)											
KIT	M541														● (blue)	● (blue)												● (blue)		
	L576P																													
	I690V																													
KRAS	Q61L																													
FOXL2	P157S																													
KIT expression		+	+	+	+	+	+	+	+	+	+	+	+	+	+	+	+	+	+	+	+	+	+	+	+	+	+	+	+	n/a

Patient No.		30	31	32	33	34	35	36	37	38	39	40	41	42	43	44	45	46	47	48	49	50	51	52	53
Stage		3	4b	4b	n/a	n/a	1	1	1	1	1	4a	4a	1	2b	2a	3	3	4b	4b	1	2a	n/a	2a	n/a
Myasthenia gravis		-	-	-	-	-	-	-	-	-	-	-	-	-	-	-	-	+	+	+	+	+	-	-	-
TET histology		THYMIC CARCINOMA					THYMOMA																		
Gene	Variant	BC	ADC	MEC	NUT	NOS	A	AB				B2					B3	B2B3					MTLS		
TP53	P72R	● (blue)	● (blue)	● (blue)	● (blue)	● (blue)	● (blue)		● (blue)	● (blue)	● (blue)	● (blue)	● (blue)	● (blue)		● (blue)	● (blue)	● (blue)	● (blue)	● (blue)	● (blue)	● (blue)	● (blue)	● (blue)	● (blue)
	G154V																								
	R158P																								
	L194H		● (red)																						
	R267fs																								
	R273C					● (red)																			
	R306*																								
	Q317*																								
ERBB2	I654V																								
	I655V								● (blue)		● (blue)				● (blue)				● (blue)		● (blue)				
	S703R																					● (orange)			
	V773M																								
KIT	M541							● (blue)											● (blue)						
	L576P	● (red)																							
	I690V																				● (orange)				
KRAS	Q61L					● (red)																			
FOXL2	P157S																							● (orange)	
KIT expression		+	-	+	-	-	-	-	-	-	-	-	-	-	-	-	-	-	-	-	-	-	-	-	-

Single nucleotide variants:

● missense variant
▼ stop gained variant
► frameshift variant

Effect on protein function (Sift) / Clinical consequence (VarSome):
tolerated / benign or likely benign (blue)
deletorius / pathogenic or likely pathogenic (red)
damaging / clinical significance uncertain (orange)

Abbreviations:

TET – Thymic epithelial tumour; SQCC - Squamous cell carcinoma; LCNEC - Large cell neuroendocrine carcinoma; BC – Basaloid carcinoma; ADC – Adenocarcinoma; MEC – Mucoepidermoid carcinoma; NUT – Nuclear protein in testis; NOS – Not otherwise specified; MTLS – Micronodular thymoma with lymphoid stroma; *TP53* – Tumour protein P53, *ERBB2* – Erb-B2 Receptor Tyrosine Kinase; *KIT* – KIT Proto-Oncogene, Receptor Tyrosine Kinase; *KRAS* – Protein V-Ki-ras2 Kirsten rat sarcoma viral oncogene homolog; *FOXL2* - Forkhead Box L2.
