# Supplementary material for: Targeted Next-Generation Sequencing of Thymic Epithelial Tumours Revealed Pathogenic Variants in KIT, ERBB2, KRAS, and TP53 in 30% of Thymic Carcinomas
Source: Cancers (Basel). 2022 Jul 12;14(14):3388. doi: 10.3390/cancers14143388 (PMC9324890; doi:10.3390/cancers14143388)
Supplement: Supplementary file 1 [file cancers-14-03388-s001.zip › Szpechcinski_Szolkowska - NGS analysis of 53 thymic epithelial tumors - Table S3.pdf]

SUPPLEMENTARY MATERIAL

Table S3. The summary of literature data on the type and frequency of *KIT* variants in patients with thymic carcinoma and preclinical or clinical efficacy of KIT tyrosine kinase inhibitors.

***KIT* mutation**		**Gene exon**	**Protein domain**	**Clinical interpretation**	**TET histology**	**Histology subtype**	**Frequency in the literature data (%)**	**Targeted treatment response**	**Reference**
**HGVSC**	**HGVSP**								
c.1468G>A	p.Glu490Lys (E490K)	9	Extracellular	Pathogenic	Thymic Carcinoma	Squamous cell carcinoma	1/17 (6%)		[1]
c.1657T>A	p.Tyr553Asn (Y553N)	11	Juxtamembrane	Pathogenic	Thymic Carcinoma	poorly differentiated	A case report	response to imatinib	[2]
					Thymic Carcinoma	n/a	1/20 (5%)	response to imatinib	[3]
c.1667_1669delTTG	p.Val556del (V556del)	11	Juxtamembrane	Uncertain	Thymic Carcinoma	Squamous cell carcinoma	1/12 (8%)		[4]
c. 1669T>C	p.Trp557Arg (W557R)	11	Juxtamembrane	Pathogenic	Thymic Carcinoma	Squamous cell carcinoma	1/17 (6%)		[1]
c. 1676T>C	p.Val559Ala (V559A)	11	Juxtamembrane	Pathogenic	Thymic Carcinoma	Squamous cell carcinoma	1/17 (6%)		[1]
					Thymic Carcinoma	n/a	1/20 (5%)		[3]
c.1676T>G	p.Val559Gly (V559G)	11	Juxtamembrane	Pathogenic	Thymic Carcinoma	Squamous cell carcinoma	A case report	response to imatinib and sunitinib	[5]
c.1679_1681del	p.Val560del (V560del)	11	Juxtamembrane	Pathogenic	Thymic Carcinoma	Epidermoid carcinoma	A case report	response to imatinib	[6]
					Thymic Carcinoma	Squamous cell carcinoma	1/7 (14%)		[7]
								sensitive to imatinib, sunitinib, dasatinib and nilotinib in vitro	[7, 8]
c.1681G>A	p.Glu561Lys (E561K)	11	Juxtamembrane	Pathogenic	Thymic Carcinoma	Squamous cell carcinoma	1/48 (2%)		[9]
c.1720_1722delACA	p.Thr574del (T574del)	11	Juxtamembrane	Uncertain	Thymic Carcinoma	Squamous cell carcinoma	1/48 (2%)		[9]
c.1724_1726del	p.Gln575del (Q575del)	11	Juxtamembrane	Uncertain	Thymic Carcinoma	Adenocarcinoma	1/48 (2%)		[9]
c.1727T>C	p.Leu576Pro (L576P)	11	Juxtamembrane	Pathogenic	Thymic Carcinoma	Squamous cell carcinoma	1/17 (6%)		[10]
								sensitive to sunitinib, imatinib, dasatinib and nilotinib in vitro	[7, 8]
					Thymic Carcinoma	Squamous cell carcinoma	1/17 (6%)		[1]
					Thymic Carcinoma	n/a	1/47 (2%)		[11]
					Thymic Carcinoma	n/a	1/20 (5%)		[3]
					Thymic Carcinoma	Squamous cell carcinoma	1/35 (3%)		[12]

c.1730_1738del	p.Pro577_Asp579del (P577_D579del)	11	Juxtamembrane	Pathogenic	Thymic Carcinoma	Squamous cell carcinoma	A case report	response to sorafenib	[13]
c.1735_1737delATG	p.Asp579del (D579del)	11	Juxtamembrane	Pathogenic	Thymic Carcinoma	Squamous cell carcinoma	A case report	response to imatinib	[14]
					Thymic Carcinoma	Squamous cell carcinoma	A case report	response to imatinib	[15]
					Thymic Carcinoma	n/a	1/47 (2%)		[11]
c.1757G>A	p.Arg586Lys (R586K)	11	Juxtamembrane	Pathogenic	Carcinoid		1/48 (2%)		[9]
c.1900C>T	p.Arg634Trp (R634W)	13	Kinase domain 1	Uncertain	Thymic Carcinoma	n/a	1/10 (10%)		[16]
c.1924A>G	p.Lys642Glu (K642E)	13	Kinase domain 1	Pathogenic	Thymic Carcinoma	Squamous cell carcinoma	A case report	response to sorafenib and imatinib	[17]
c.2089C>T	p.His697Tyr (H697Y)	14	Kinase insert	Pathogenic	Thymic Carcinoma	Squamous cell carcinoma	1/7 (14%)	sensitive to sunitinib in vitro	[7]
c.2460T>A	p.Asp820Glu (D820E)	17	Distal kinase	Pathogenic				sensitive to dasatinib and nilotinib in vitro	[7, 8]
					Thymic Carcinoma	undifferentiated	A case report	response to sorafenib	[18]
					Thymic Carcinoma	Squamous cell carcinoma	1/17 (6%)		[1]
					Thymic Carcinoma	Neuroendocrine	A case report	response to sorafenib and sunitinib	[19]
					Thymic Carcinoma	n/a	1/20 (5%)	response to sorafenib	[3]
c.2468A>C	p.Tyr823Ser (Y823S)	17	Distal kinase	Uncertain	Thymic Carcinoma	Squamous cell carcinoma	1/35 (3%)		[12]
c.2515G>A	p.Glu839Lys (E839K)	18	Kinase domain 2	Pathogenic	Thymic Carcinoma	n/a	1/10 (10%)		[16]

References

[1] Schirosi L, Nannini N, Nicoli D, Cavazza A, Valli R, Buti S, et al. Activating c-KIT mutations in a subset of thymic carcinoma and response to different c-KIT inhibitors. Ann Oncol. 2012;23:2409-14.

[2] Buti S, Donini M, Sergio P, Garagnani L, Schirosi L, Passalacqua R, et al. Impressive response with imatinib in a heavily pretreated patient with metastatic c-KIT mutated thymic carcinoma. J Clin Oncol. 2011;29:e803-5.

[3] Tiseo M, Damato A, Longo L, Barbieri F, Bertolini F, Stefani A, et al. Analysis of a panel of druggable gene mutations and of ALK and PD-L1 expression in a series of thymic epithelial tumors (TETs). Lung Cancer. 2017;104:24-30.

[4] Shitara M, Okuda K, Suzuki A, Tatematsu T, Hikosaka Y, Moriyama S, et al. Genetic profiling of thymic carcinoma using targeted next-generation sequencing. Lung Cancer. 2014;86:174-9.

[5] Hirai F, Edagawa M, Shimamatsu S, Toyozawa R, Toyokawa G, Nosaki K, et al. c-kit mutation-positive advanced thymic carcinoma successfully treated as a mediastinal gastrointestinal stromal tumor: A case report. Mol Clin Oncol. 2016;4:527-9.
[6] Strobel P, Hartmann M, Jakob A, Mikesch K, Brink I, Dirnhofer S, et al. Thymic carcinoma with overexpression of mutated KIT and the response to imatinib. N Engl J Med. 2004;350:2625-6.
[7] Girard N, Shen R, Guo T, Zakowski MF, Heguy A, Riely GJ, et al. Comprehensive genomic analysis reveals clinically relevant molecular distinctions between thymic carcinomas and thymomas. Clin Cancer Res. 2009;15:6790-9.
[8] Girard N. Thymic tumors: relevant molecular data in the clinic. J Thorac Oncol. 2010;5:S291-5.
[9] Sakane T, Sakamoto Y, Masaki A, Murase T, Okuda K, Nakanishi R, et al. Mutation Profile of Thymic Carcinoma and Thymic Neuroendocrine Tumor by Targeted Next-generation Sequencing. Clin Lung Cancer. 2021;22:92-9 e4.
[10] Yoh K, Nishiwaki Y, Ishii G, Goto K, Kubota K, Ohmatsu H, et al. Mutational status of EGFR and KIT in thymoma and thymic carcinoma. Lung Cancer. 2008;62:316-20.
[11] Wang Y, Thomas A, Lau C, Rajan A, Zhu Y, Killian JK, et al. Mutations of epigenetic regulatory genes are common in thymic carcinomas. Sci Rep. 2014;4:7336.
[12] Enkner F, Pichlhofer B, Zaharie AT, Krunic M, Holper TM, Janik S, et al. Molecular Profiling of Thymoma and Thymic Carcinoma: Genetic Differences and Potential Novel Therapeutic Targets. Pathol Oncol Res. 2017;23:551-64.
[13] Disel U, Oztuzcu S, Besen AA, Karadeniz C, Kose F, Sumbul AT, et al. Promising efficacy of sorafenib in a relapsed thymic carcinoma with C-KIT exon 11 deletion mutation. Lung Cancer. 2011;71:109-12.
[14] Hagemann IS, Govindan R, Javidan-Nejad C, Pfeifer JD, Cottrell CE. Stabilization of disease after targeted therapy in a thymic carcinoma with KIT mutation detected by clinical next-generation sequencing. J Thorac Oncol. 2014;9:e12-6.
[15] Lim SH, Lee JY, Sun JM, Kim KM, Ahn JS, Ahn MJ, et al. A new KIT gene mutation in thymic cancer and a promising response to imatinib. J Thorac Oncol. 2013;8:e91-2.
[16] Casini B, Sarti D, Gallo E, Alessandrini G, Cecere F, Pescarmona E, et al. Thymic carcinoma: preliminary data of next generation sequencing analysis. Mediastinum; 2018. p. AB008.
[17] Catania C, Conforti F, Spitaleri G, Barberis M, Preda L, Noberasco C, et al. Antitumor activity of sorafenib and imatinib in a patient with thymic carcinoma harboring c-KIT exon 13 missense mutation K642E. Onco Targets Ther. 2014;7:697-702.
[18] Bisagni G, Rossi G, Cavazza A, Sartori G, Gardini G, Boni C. Long lasting response to the multikinase inhibitor bay 43-9006 (Sorafenib) in a heavily pretreated metastatic thymic carcinoma. J Thorac Oncol. 2009;4:773-5.
[19] Pagano M, Sierra NM, Panebianco M, Rossi G, Gnoni R, Bisagni G, et al. Sorafenib efficacy in thymic carcinomas seems not to require c-KIT or PDGFR-alpha mutations. Anticancer Res. 2014;34:5105-10.
